
JOURNAL INFORMATION
==============================
NLM Title Abbreviation: ScientificWorldJournal
Journal ID: ScientificWorldJournal
NLM Journal ID: 101131163
Journal ID: TSWJ

The Scientific World Journal

ISSN: 2356-6140
EISSN: 1537-744X
Publisher: Wiley

ARTICLE INFORMATION
==============================
PMCID: PMC6009265
PMID: 29973799
DOI: 10.1100/tsw.2002.25
Article ID: 325768
Article version: 1
Subjects: Short Report

The Protein Trinity: Structure/Function Relationships That Include Intrinsic Disorder

Dunker A. Keith 1 *
Brown Celeste J. 1
Lawson Celeste J. David 1
Iakoucheva-Sebat Lilia M. 1
Vucetic Slobodan 2
Obradovic Zoran 2
School of Molecular Biosciences, Washington State University, Pullman, WA 99164-4660, USA
Center for Information Sciences Temple University Philadelphia, PA 19122, USA
*A. Keith Dunker: dunker@mail.wsu.edu
Publication date: 2002
Electronic publication date: 2002 Jun 25
Volume: 2
First page: 49
Last page: 50
Copyright: Copyright © 2002 A. Keith Dunker et al.
Copyright year: 2002
License: This is an open access article distributed under the Creative Commons Attribution License, which permits unrestricted use, distribution, and reproduction in any medium, provided the original work is properly cited.
License URL: https://creativecommons.org/licenses/by/3.0/

==============================
Advances in Gene Technology: The Genome and Beyond –
Structural Biology for Medicine (Proceedings of the 2002 Miami
*Nature Biotechnology* Winter Symposium)
*TheScientificWorld* 2002, 2(S2), 49–50
ISSN 1532-2246; DOI 10.1100/tsw.2002.25

### THE PROTEIN TRINITY: STRUCTURE/FUNCTION RELATIONSHIPS THAT INCLUDE INTRINSIC DISORDER

A. Keith Dunker[1,]*, Celeste J. Brown[1], J. David Lawson[1], Lilia M. Iakoucheva-Sebat[1], Slobodan Vucetic[2], and Zoran Obradovic[2]
[1]School of Molecular Biosciences, Washington State University, Pullman, WA 99164-4660 and [2]Center for Information Sciences Temple University Philadelphia, PA 19122
*Corresponding author: dunker@mail.wsu.edu

**INTRODUCTION.** The current, dominant view relating protein structure to function can be expressed as amino acid sequence → 3-D structure → function. Currently, more than 200 counter examples in which function depends on nonfolded or incompletely folded regions of protein have been described. Furthermore, reviews on intrinsically disordered proteins are beginning to appear[1,2,3]. In one of these reviews, it is suggested that the existence of proteins with intrinsic protein disorder calls for a re-assessment of the protein structure-function paradigm[3].

**METHODOLOGY.** From literature and database searches, we collected a set of proteins that are structurally characterized to have regions of disorder or to be wholly disordered under physiological conditions. Once our database was assembled, we used various computational techniques to construct predictors of natural disordered regions (PONDRs).

| Protein | LDR/Protein Size | Function |
|---|---|---|
| c-Jun | 35/331 | DNA binding |
| FlgM | 97/97 | Protein binding, Channel transport |
| 4E-BP1 | 118/118 | Protein binding, Phosphorylation |
| Neurofilament H | 679/1087 | Entropic bristle, Phosphorylation, Gycoslyation |
| Titin | 2174/~33,000 | Entropic spring |

**TABLE 1.** Representative samples from our database of over 90 nonhomologous proteins that contain long disordered regions (LDRs) of ≥30 contiguous amino acids. "LDR/Protein Size" represents the length of the disordered region and the protein respectively. "Function" refers to the function of the disordered region.

| Kingdom | # of Proteomes | Disorder |
|---|---|---|
| Prokaryia | 22 | 7–33% |
| Archaea | 7 | 9–37% |
| Eukaryia | 5 | 36–63% |

**TABLE 2**. Each proteome from the three kingdoms was scanned for disorder using a PONDR predictor. The score for each proteome represents the percentage of the proteins in each proteome predicted to contain LDRs of ≥40 contiguous amino acids. The disorder scores for each kingdom are reported as the range of disorder observed throughout all of the proteomes in each kingdom.

**CONCLUSIONS.** Using the PONDR predictors, we were able to confirm the hypothesis that, just as amino acid sequence determines 3-D structure, it also determines the lack of 3-D structure. Results from these predictors further suggest that the most common type of protein in eukaryotic cells contains both ordered and intrinsically disordered regions. This is likely due to the large amount of signaling proteins in eukaryotic cells. Given that proteins with intrinsically disordered regions are so common, the model relating protein structure to function needs to be reassessed. Here, to accommodate intrinsic disorder, we propose The Protein Trinity (Fig. 1). Following Ptitsyn[4], this proposal envisions three distinct protein states, described approximately as the ordered state, the collapsed state and the extended state. According to The Protein Trinity, any one of these three forms (not just the ordered state) can exist in native proteins.

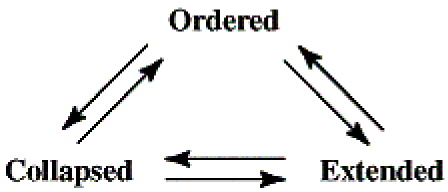

**FIGURE 1.** The Protein Trinity.

**REFERENCES**

1. Dunker, A.K., Lawson, J.D., Brown, C.J., Williams, R.M., Romero, P., Oh, J.S., Oldfield, C.J., Campen, A.M., Ratliff, C.M., Hipps, K.W., Ausio, J., Nissen, M.S., Reeves, R., Kang, C.H., Kissinger, C.R., Bailey, R.W., Griswold, M.D., Chiu, W., Garner, E.C., and Obradovic, Z. (2001) J. Mol. Graph. Model. 19, 26–59.
2. Namba, K. (2001) Genes Cells 6, 1–12.
3. Wright, P.E. and Dyson, H.J. (1999) J. Mol. Biol. 293, 321–331.
4. Ptitsyn, O.B. and Uversky, V.N. (1994) FEBS Lett. 341, 15–18.
